# Risk of 16 cancers across the full glycemic spectrum: a population-based cohort study using the UK Biobank

**DOI:** 10.1136/bmjdrc-2020-001600

**Published:** 2020-08-27

**Authors:** Christopher T Rentsch, Ruth E Farmer, Sophie V Eastwood, Rohini Mathur, Victoria Garfield, Aliki-Eleni Farmaki, Krishnan Bhaskaran, Nish Chaturvedi, Liam Smeeth

**Affiliations:** 1 Department of Non-Communicable Disease Epidemiology, Faculty of Epidemiology and Population Health, London School of Hygiene & Tropical Medicine, London, UK; 2 MRC Unit for Lifelong Health and Ageing at UCL, Institute of Cardiovascular Science, University College London, London, UK

**Keywords:** glycated hemoglobin A, hyperglycemia, cohort studies, epidemiology

## Abstract

**Introduction:**

Diabetes is observed to increase cancer risk, leading to hypothesized direct effects of either hyperglycemia or medication. We investigated associations between glycosylated hemoglobin (HbA1c) across the whole glycemic spectrum and incidence of 16 cancers in a population sample with comprehensive adjustment for risk factors and medication.

**Research design and methods:**

Linked data from the UK Biobank and UK cancer registry for all individuals with baseline HbA1c and no history of cancer at enrollment were used. Incident cancer was based on International Classification of Diseases – 10th Edition diagnostic codes. Age-standardized incidence rates were estimated by HbA1c category. Associations between HbA1c, modeled as a restricted cubic spline, and cancer risk were estimated using Cox proportional hazards models.

**Results:**

Among 378 253 individuals with average follow-up of 7.1 years, 21 172 incident cancers occurred. While incidence for many of the 16 cancers was associated with hyperglycemia in crude analyses, these associations disappeared after multivariable adjustment, except for pancreatic cancer (HR 1.55, 95% CI 1.22 to 1.98 for 55 vs 35 mmol/mol), and a novel finding of an inverse association between HbA1c and premenopausal breast cancer (HR 1.27, 95% CI 1.00 to 1.60 for 25 vs 35 mmol/mol; HR 0.71, 95% CI 0.54 to 0.94 for 45 vs 35 mmol/mol), not observed for postmenopausal breast cancer. Adjustment for diabetes medications had no appreciable impact on HRs for cancer.

**Conclusions:**

Apart from pancreatic cancer, we did not demonstrate any independent positive association between HbA1c and cancer risk. These findings suggest that the potential for a cancer-inducing, direct effect of hyperglycemia may be misplaced.

**Significance of this study**

**What is already known about this subject?**

- Diabetes has been associated with increased cancer risk.

**What are the new findings?**

- This study is the largest to date to investigate the association between glycosylated hemoglobin (HbA1c) and 16 specific cancers adjusting for a wide range of demographic, lifestyle, and clinical factors.
- We found no consistent evidence that higher HbA1c was associated with incident cancer risk apart from pancreatic cancer.
- A novel finding was an inverse association between HbA1c and premenopausal breast cancer, which persisted when people with diagnosed diabetes or on glucose-lowering medications were excluded.

**How might these results change the focus of research or clinical practice?**

- Concerns around the potential for a cancer-inducing, direct effect of hyperglycemia may be misplaced.
- Future research should focus on other potential mechanisms that may explain the association between diabetes and cancer.

## Introduction

Multiple observational studies have shown an increased risk of many cancer types in people with diabetes, independent of body mass index (BMI).^1 2^ The reasons for this are not fully understood but may include direct effects of hyperglycemia, insulin resistance, genetic risk, chronic inflammation or other associated metabolic abnormalities.^3^ Alternatively, effects of glucose-lowering medication may account for excess risks of cancer in association with diabetes.^3^

Studying cancer risk across the entire glycemic spectrum may help distinguish between medication and underlying disease-related effects. Recent systematic reviews are conflicting, postulating positive, U-shaped and null associations between glycosylated hemoglobin (HbA1c) and overall cancer incidence.^4 5^ Such conflicting findings may, in part, be due to small numbers, especially for those without diabetes. Furthermore, relationships between HbA1c and cancer may differ by cancer site.^6^ More recently, Dankner *et al*^7^ studied 440 000 individuals with type 2 diabetes, including over 20 000 incident cancer cases during follow-up. The authors observed no evidence of association between poor glycemic control and cancer risk except for an adverse effect on pancreatic cancer and a protective effect for prostate cancer.

Large numbers of individuals without diabetes or not considered to be at high risk for diabetes are unlikely to have measurements of glycemic status such as HbA1c in routine care. However, UK Biobank collected HbA1c measurements at recruitment on ~500 000 participants, regardless of diabetes status, presenting a unique opportunity to study associations with health across the glycemic spectrum. We studied the association between HbA1c and incidence of 16 site-specific cancers within UK Biobank, both in the overall cohort and in those without a diabetes diagnosis, to help disentangle the effects of glycemic status from any effect(s) of glucose-lowering medications.

## Design and study methods

### Study population

Full details of the UK Biobank cohort have been previously described.^8 9^ Briefly, the UK Biobank includes 502 536 men and women aged 40–69 years recruited between 2006 and 2010 from primary care practices in England, Scotland, and Wales. Participants underwent a baseline assessment capturing sociodemographic and lifestyle factors, health status, and gave blood samples for biomarker measurement. Participants also consented for linkage to electronic medical records including national cancer and death registries. All participants were eligible for this study. We excluded individuals with a history of any cancer (excluding non-melanoma skin cancer (NMSC)) at baseline, either via self-report at baseline assessment under consultation with a trained nurse or via linked cancer registry records.

### Study period

Baseline for this study was defined as the date of baseline assessment. Participants were considered at risk from 6 months after baseline to reduce the impact of reverse causality among cancers diagnosed shortly after baseline, whereby the undiagnosed cancer may have affected the baseline HbA1c measurement. Follow-up ended at the earliest of: first record of any cancer (as defined below), last date of coverage for the cancer registry (March 2016 in England and Wales; October 2015 in Scotland), or date of death. For premenopausal breast cancer outcomes, participants were additionally censored at age 55 years.

### Exposure

HbA1c (mmol/mol) was measured using high-performance liquid chromatography from blood samples taken at baseline. A total of 33 104 (7%) of participants had missing values returned, with 14 565 (44%) of these related to assay failure or dilution factors. Values above 200 mmol/mol were considered outliers and excluded from the analysis.

### Cancer outcomes

Cancer incidence was defined as the first occurrence of an International Classification of Diseases – 10th Edition (ICD-10) code for malignant cancer (ICD-10 Chapter C) excluding NMSC (C44) from the cancer registry. We also considered deaths for which cancer was a primary or contributing cause of death as incident cancer if there was no preceding record in the cancer registry.

The four most common cancers in the cohort were considered primary outcomes of interest, including prostate (C61), breast (C50), colorectal (C18–C20) and lung (C34). Breast cancers were analyzed separately by menopausal status. Postmenopausal status was defined as either age 55 years or older, or taking hormone replacement therapy (HRT), consistent with previous literature.^10^ We also examined 12 secondary cancer outcomes that were less common in the cohort: esophageal (C15), stomach (C16), pancreatic cancer (C25), melanoma (C43), uterus (C54–55), ovarian (C56), kidney (C64), bladder (C67), central nervous system (C71–72), non-Hodgkin’s lymphoma (C82–85), multiple myeloma (C90), and leukemia (C91–94).

### Covariates

We extracted the following variables from the baseline assessment data: demographic and socioeconomic factors (age, sex, ethnicity and Townsend deprivation index); BMI; physical activity (number of days per week engaging in more than 10 min of walking, moderate and vigorous physical activity); lifestyle behaviors (smoking status and alcohol consumption); dietary intake (processed meat, fruits and vegetables); use of medications (such antidiabetic therapy, HRT, and oral contraceptives); and diagnoses of comorbidities (cardiovascular disease and types 1 and 2 diabetes). Rate of smoking among current or former smokers was categorized as 1–9, 10–19, 20–29, and 30+ cigarettes smoked per day. We used a previously validated algorithm to identify diagnosed types 1 and 2 diabetes, which included exposure to glucose-lowering therapies.^11^

**Figure 1:**
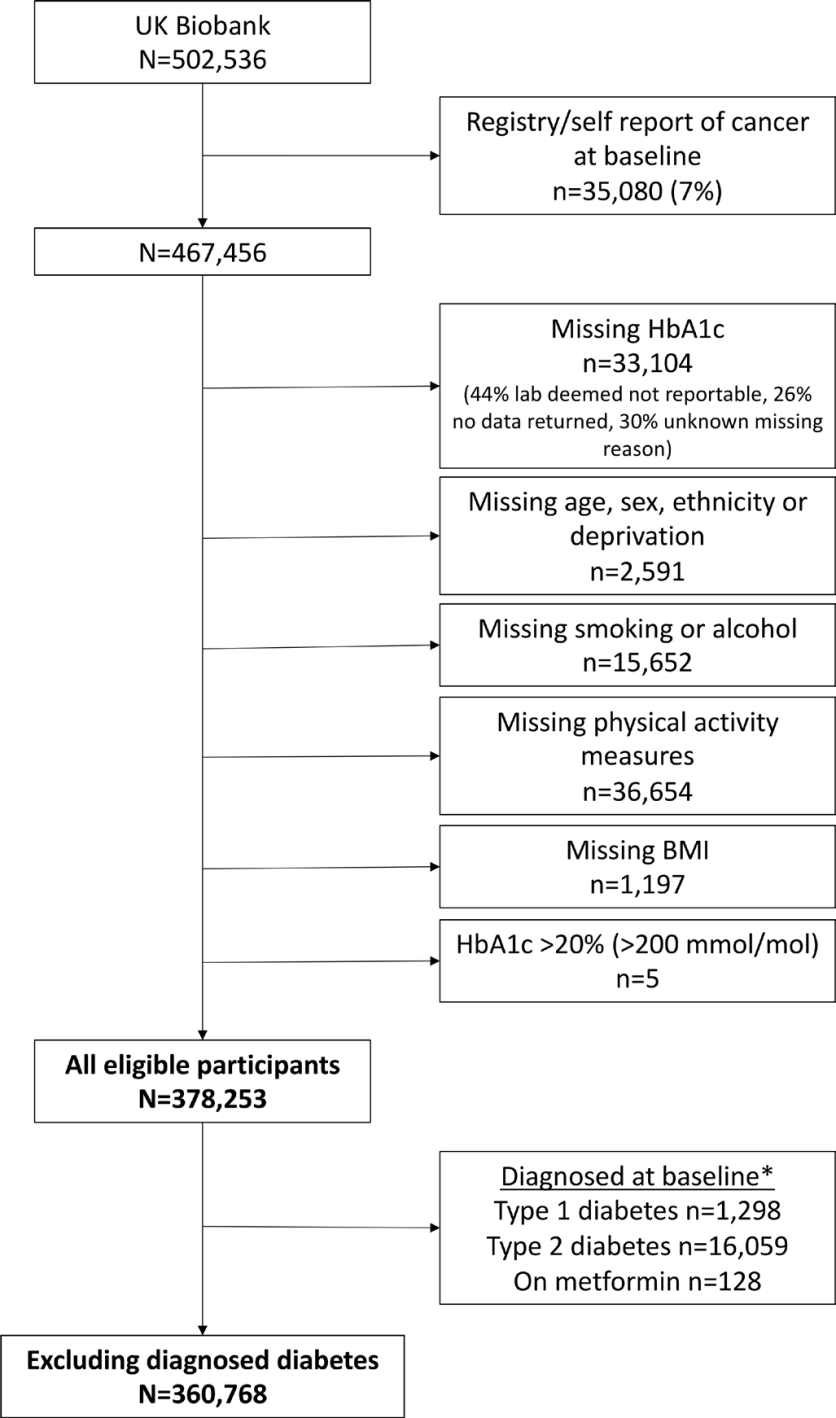
Study flow chart. *We used a previously validated algorithm to identify diagnosed type 1 and 2 diabetes, which included exposure to glucose-lowering therapies. A small number of individuals (n=128) on metformin for other indications were also excluded from some modeling. BMI, body mass index; HbA1c, glycated hemoglobin.

Among 467 456 participants eligible for study inclusion, 89 203 (19%) had missing baseline data. A large proportion of missingness was driven by lack of baseline HbA1c (n=33 104, 7%), self-reported physical activity measures (n=36 654, 8%), and self-reported smoking or alcohol measures (n=15 652, 3%) (figure 1). Reasons for missing baseline HbA1c included laboratory reporting and data issues (70%) and therefore largely missing at random, while the remaining 30% were missing for some unknown reason. Lifestyle measures were missing because participants responded, ‘prefers not to say’, thus likely missing not at random.

### Statistical analysis

For descriptive analyses of covariates and cancer incidence rates, HbA1c was categorized at standard clinical cut-off points for normal glycemia (<6% or <42 mmol/mol), prediabetes (6%–6.4% or 42–47 mmol/mol) and diabetes (≥6.5% or ≥48 mmol/mol), irrespective of diagnosed diabetes status at recruitment. Incidence rates for each cancer were estimated by HbA1c category and age standardized to the UK Biobank population. Percentile CIs were estimated using 500 bootstrap replications since traditional methods are unable handle non-integer denominators.

For all other analyses, HbA1c was treated as a continuous variable modeled as a restricted cubic spline with knots at 4.4%, 5.4%, and 6.3% (25, 35, and 45 mmol/mol). We centred these parameters around 5.4% (35 mmol/mol)—the median value for those in the normal glycemia category—to provide a relevant reference value. HRs and 95% CI for the effect of HbA1c on cancer incidence were estimated using Cox proportional hazards models using time since baseline as the time scale. Likelihood ratio tests were used to assess the overall effect of HbA1c and any evidence of non-linearity. The proportional hazards assumption was tested by inspecting whether scaled Schoenfeld residuals were independent of time.

All models were adjusted for the available known risk factors for cancer that are also likely to influence HbA1c, namely, age, sex (except sex-specific cancers), ethnicity, deprivation, BMI, physical activity, cardiovascular and diabetes diagnoses at baseline, smoking status, and alcohol consumption. Analyses of breast, uterus, and ovarian cancers were additionally adjusted for use of HRT (postmenopausal only) and oral contraceptives. Analyses of colorectal and stomach cancers were additionally adjusted for dietary intake. Analyses of lung, esophageal, and stomach cancers were additionally adjusted for age at start and stop of smoking among former smokers and rate of smoking among current smokers. All models were then refitted on the sample restricted to those without a diagnosis of diabetes at baseline to examine the effect of HbA1c in the absence of long-term exposure to glucose-lowering medications. A small number of individuals (n=128) on metformin for other indications were also excluded.

We used complete case analysis because a large proportion of missingness was likely to be missing not at random. In this circumstance, although multiple imputation is not appropriate, a complete case analysis will be unbiased if, conditional on model covariates, missingness is independent of the outcome.^12^

### Sensitivity analyses

First, we investigated whether the effect of HbA1c on risk of cancer differed in a subgroup without smoking history, a key risk factor for many of the included cancer outcomes. Second, we assessed the potential for reverse causality by comparing the association between HbA1c and cancer incidence by time since study entry (0–6 months, 6 months–2 years, and 2+ years) for the primary outcomes. Third, we assessed changes in the association between HbA1c and risk of the primary cancer outcomes at each stage of confounder adjustment to identify which variables appeared to have greater impact on the observed results. Finally, we estimated the association between having a type 2 diabetes diagnosis at baseline and risk of cancer incidence, not adjusting for HbA1c, to determine if we could replicate established associations from multiple previous studies.^2^ If similar associations were observed to those reported previously, then this would provide reassurance that potential null findings for the effect of HbA1c were unlikely to be explained by a relatively healthier population as has been found in UK Biobank compared with the general population.^13^ In post hoc analyses, we adjusted estimates for premenopausal breast cancer with maternal age at first live birth, which was available on 60% of the modeled sample. We also tested the robustness of the choice of timescale by comparing estimates from primary analyses to those from models using age as the timescale.

## Results

### Cohort description

Of the 502 536 participants in the UK Biobank, 35 080 were excluded for having a history of cancer at the baseline assessment. A further 89 203 were excluded for having missing data on at least one of the covariates of interest, leaving a total of 378 253 participants eligible to contribute to analysis (figure 1). Of these, 349 825 (93%) had normal, 15 648 (4%) had prediabetic and 12 780 (3%) had diabetic levels of HbA1c at baseline. Individuals with higher HbA1c (prediabetic or diabetic) were observed to have higher median BMI, were less likely to do moderate or vigorous physical activity, were more deprived and more likely to be of non-white ethnicity compared with those with normal HbA1c (table 1). Of the 378 253 eligible participants, 17 485 (5%) reported a diabetes diagnoses or were on metformin at baseline. Characteristics of the 360 768 participants without diagnosed diabetes or on metformin at baseline are provided in online supplementary table S1.

10.1136/bmjdrc-2020-001600.supp1 Supplementary data

**Table 1:** Baseline characteristics of 378 253 eligible UK Biobank participants

	<6% or <42 mmol/mol	6%–6.4% or 42–47 mmol/mol	≥6.5% or ≥48 mmol/mol
N	349 825	15 648	12 780
Age at baseline assessment, years (median (IQR))	57.0 (49.0 to 63.0)	61.0 (56.0 to 65.0)	61.0 (54.0 to 65.0)
Female (N %)	189 945 (54.3)	7401 (47.3)	4644 (36.3)
Ethnicity			
White European	336 172 (96.1)	13 812 (88.3)	11 288 (88.3)
South Asian	3845 (1.1)	590 (3.8)	627 (4.9)
African Caribbean	3535 (1.0)	639 (4.1)	399 (3.1)
Mixed or other	6273 (1.8)	607 (3.9)	466 (3.6)
Townsend deprivation index quintile			
Least deprived	74 442 (21.3)	2693 (17.2)	1987 (15.5)
Second least deprived	73 142 (20.9)	2853 (18.2)	2162 (16.9)
Median deprivation level	71 721 (20.5)	2914 (18.6)	2337 (18.3)
Second most deprived	69 056 (19.7)	3309 (21.1)	2693 (21.1)
Most deprived	61 464 (17.6)	3879 (24.8)	3601 (28.2)
Smoking status			
Never smoker	245 290 (70.1)	9109 (58.2)	7417 (58.0)
Former smoker	80 703 (23.1)	4786 (30.6)	4273 (33.4)
1–9 CPD	5144 (1.5)	240 (1.5)	141 (1.1)
10–19 CPD	10 107 (2.9)	750 (4.8)	453 (3.5)
20–29 CPD	6814 (1.9)	580 (3.7)	355 (2.8)
30+ CPD	1767 (0.5)	183 (1.2)	141 (1.1)
Alcohol use			
Daily or almost daily	74 105 (21.2)	2562 (16.4)	1853 (14.5)
Three or four times a week	86 080 (24.6)	2682 (17.1)	1965 (15.4)
Once or twice a week	91 703 (26.2)	3851 (24.6)	3063 (24.0)
One to three times a month	38 642 (11.0)	1991 (12.7)	1684 (13.2)
Special occasions only	35 760 (10.2)	2634 (16.8)	2288 (17.9)
Never	23 535 (6.7)	1928 (12.3)	1927 (15.1)
Days per week spent doing moderate physical activity			
None	43 485 (12.4)	2573 (16.4)	2585 (20.2)
1–2	80 980 (23.1)	3329 (21.3)	2812 (22.0)
3–4	53 350 (15.3)	2278 (14.6)	1766 (13.8)
5+	172 010 (49.2)	7468 (47.7)	5617 (44.0)
Days per week spent doing vigorous physical activity			
None	122 655 (35.1)	7073 (45.2)	6287 (49.2)
1–2	108 371 (31.0)	4164 (26.6)	3158 (24.7)
3–4	50 518 (14.4)	1772 (11.3)	1319 (10.3)
5+	68 281 (19.5)	2639 (16.9)	2016 (15.8)
Days per week walked for >10 min			
None	7287 (2.1)	504 (3.2)	592 (4.6)
1–2	31 086 (8.9)	1523 (9.7)	1279 (10.0)
3–4	27 730 (7.9)	1269 (8.1)	1103 (8.6)
5+	283 722 (81.1)	12 352 (78.9)	9806 (76.7)
Days per week with intake of processed meats			
None	32 903 (9.4)	1161 (7.4)	837 (6.6)
<1	108 194 (30.9)	4294 (27.4)	3053 (23.9)
1	101 933 (29.1)	4626 (29.6)	3744 (29.3)
2–4	93 383 (26.7)	4822 (30.8)	4390 (34.4)
5+	13 412 (3.8)	745 (4.8)	756 (5.9)
Number of fruits or vegetables consumed per day			
None	10 712 (3.1)	685 (4.4)	501 (3.9)
1–2	61 487 (17.6)	2831 (18.1)	2325 (18.2)
3–4	102 465 (29.3)	4527 (28.9)	3742 (29.3)
5+	175, 161 (50.1)	7605 (48.6)	6212 (48.6)
BMI at baseline, kg/m^2^ (median (IQR))	26.4 (23.9 to 29.4)	29.7 (26.7 to 33.5)	30.8 (27.5 to 34.9)
HbA1c at baseline, mmol/mol (median (IQR))	34.7 (32.4 to 37.0)	43.7 (42.7 to 45.2)	56.6 (51.4 to 65.3)
Comorbidities reported at baseline			
Any cardiovascular disease	40 841 (11.7)	3660 (23.4)	3446 (27.0)
Type 1 diabetes	43 (0.0)	112 (0.7)	1143 (8.9)
Type 1 diabetes	3277 (0.9)	3693 (23.6)	9089 (71.1)
Medications reported at baseline			
Metformin	1258 (0.4)	2049 (13.1)	6611 (51.7)
Other oral antidiabetic therapy	409 (0.1)	824 (5.3)	3312 (25.9)
Insulin	257 (0.1)	381 (2.4)	3107 (24.3)
Hormone replacement therapy	14 517 (4.2)	321 (2.1)	222 (1.7)
Oral contraceptives	5631 (1.6)	60 (0.4)	92 (0.7)

BMI, body mass index in kilograms per square metre (kg/m^2^); CPD, cigarettes smoked per day; HbA1c, glycated hemoglobin.

### Cancer incidence

Among 378 253 individuals with median follow-up time of 7.1 years (IQR 6.4–7.7 years) for a total 2 425 635 person-years (PY), 21 172 (5.6%) had an incident cancer diagnosis. Age-standardized rates per 1000 PY for any cancer in those with normal, prediabetic, and diabetic levels of HbA1c were 8.65 (95% CI 8.54 to 8.77), 10.69 (95% CI 10.04 to 11.28), and 9.89 (95% 9.23 to 10.49), respectively (table 2). Age-standardized rates per 1000 PY of prostate cancer were lower among individuals with diabetic levels of HbA1c (2.84, 95% CI 2.44 to 3.24) than those with normal HbA1c (4.09, 95% CI 3.96 to 4.21). Conversely, rates of colorectal cancer per 1000 PY were higher among individuals with diabetic levels of HbA1c (1.34, 95% CI 1.07 to 1.62) than those with normal HbA1c (1.01, 95% CI 0.97 to 1.05). For lung and postmenopausal breast cancer, rates were similar between those with normal and diabetic levels of HbA1c but elevated among those with prediabetic levels of HbA1c. Similar rates of premenopausal breast cancer were observed among those with normal and diabetic levels of HbA1c but lower among those with prediabetic levels of HbA1c; however, there were only five cases in this group. Incidence rates did not meaningfully change among patients without a baseline diabetes diagnosis or exposure to metformin (online supplementary table S2) or rates including the first 6 months after baseline (online supplementary table S3).

**Table 2:** Number of events and age-standardized incidence rates in the UK Biobank population for 16 different cancers by HbA1c category

	<6% or <42 mmol/mol		6%–6.4% or 42–47 mmol/mol		≥6.5% or ≥48 mmol/mol	
	N	Rate/1000 PY (95% CI)	N	Rate/1000 PY (95% CI)	N	Rate/1000 PY (95% CI)
Any malignant cancer*	19 005	8.65 (8.54 to 8.77)	1256	10.69 (10.04 to 11.28)	911	9.89 (9.23 to 10.49)
Primary outcomes						
Prostate (C61) †	4108	4.09 (3.96 to 4.21)	238	3.64 (3.11 to 4.13)	181	2.84 (2.44 to 3.24)
Premenopausal breast (C50) ‡	1006	1.11 (1.04 to 1.19)	5	0.38 (0.07 to 0.75)	13	1.09 (0.54 to 1.67)
Postmenopausal breast (C50) ‡	2595	3.34 (3.08 to 3.62)	134	6.82 (1.96 to 16.07)	84	3.04 (1.92 to 4.92)
Colorectal (C18–C20)	2207	1.01 (0.97 to 1.05)	151	1.27 (1.04 to 1.49)	118	1.34 (1.07 to 1.62)
Lung (C34)	1377	0.63 (0.60 to 0.67)	164	1.37 (1.12 to 1.59)	81	0.85 (0.66 to 1.05)
Secondary outcomes						
Esophageal (C15)	394	0.18 (0.17 to 0.20)	39	0.31 (0.22 to 0.42)	40	0.43 (0.32 to 0.57)
Stomach (C16)	274	0.13 (0.11 to 0.14)	33	0.25 (0.17 to 0.35)	20	0.25 (0.15 to 0.37)
Pancreatic (C25)	497	0.23 (0.21 to 0.25)	49	0.46 (0.33 to 0.63)	54	0.59 (0.43 to 0.76)
Melanoma (C43)	1172	0.53 (0.50 to 0.56)	63	0.52 (0.39 to 0.66)	40	0.43 (0.30 to 0.56)
Uterus (C54–55) ‡	563	0.47 (0.43 to 0.51)	49	1.00 (0.72 to 1.34)	29	0.89 (0.59 to 1.22)
Ovarian (C56) ‡	439	0.37 (0.34 to 0.41)	17	0.40 (0.18 to 0.62)	15	0.48 (0.25 to 0.75)
Kidney (C64)	504	0.23 (0.21 to 0.25)	42	0.39 (0.27 to 0.51)	41	0.44 (0.31 to 0.56)
Bladder (C67)	399	0.19 (0.17 to 0.20)	30	0.21 (0.14 to 0.29)	40	0.42 (0.30 to 0.56)
Central nervous system (C71–72)	366	0.17 (0.15 to 0.18)	27	0.27 (0.17 to 0.38)	14	0.16 (0.08 to 0.25)
Non-Hodgkin’s lymphoma (C82–85)	830	0.38 (0.36 to 0.41)	62	0.49 (0.35 to 0.61)	38	0.44 (0.32 to 0.59)
Multiple myeloma (C90)	319	0.15 (0.13 to 0.16)	19	0.15 (0.08 to 0.22)	17	0.18 (0.11 to 0.26)
Leukemia (C91–94)	485	0.22 (0.20 to 0.24)	42	0.23 (0.10 to 0.39)	23	0.13 (0.03 to 0.25)

Standardized to the UK Biobank population, percentile CIs were obtained via 500 bootstrap replications.

*Excluding non-melanoma skin cancer (C44).

†Males only.

‡Females only.

HbA1c, glycated hemoglobin; PY, person-years.

### Adjusted analyses

**Figure 2:**
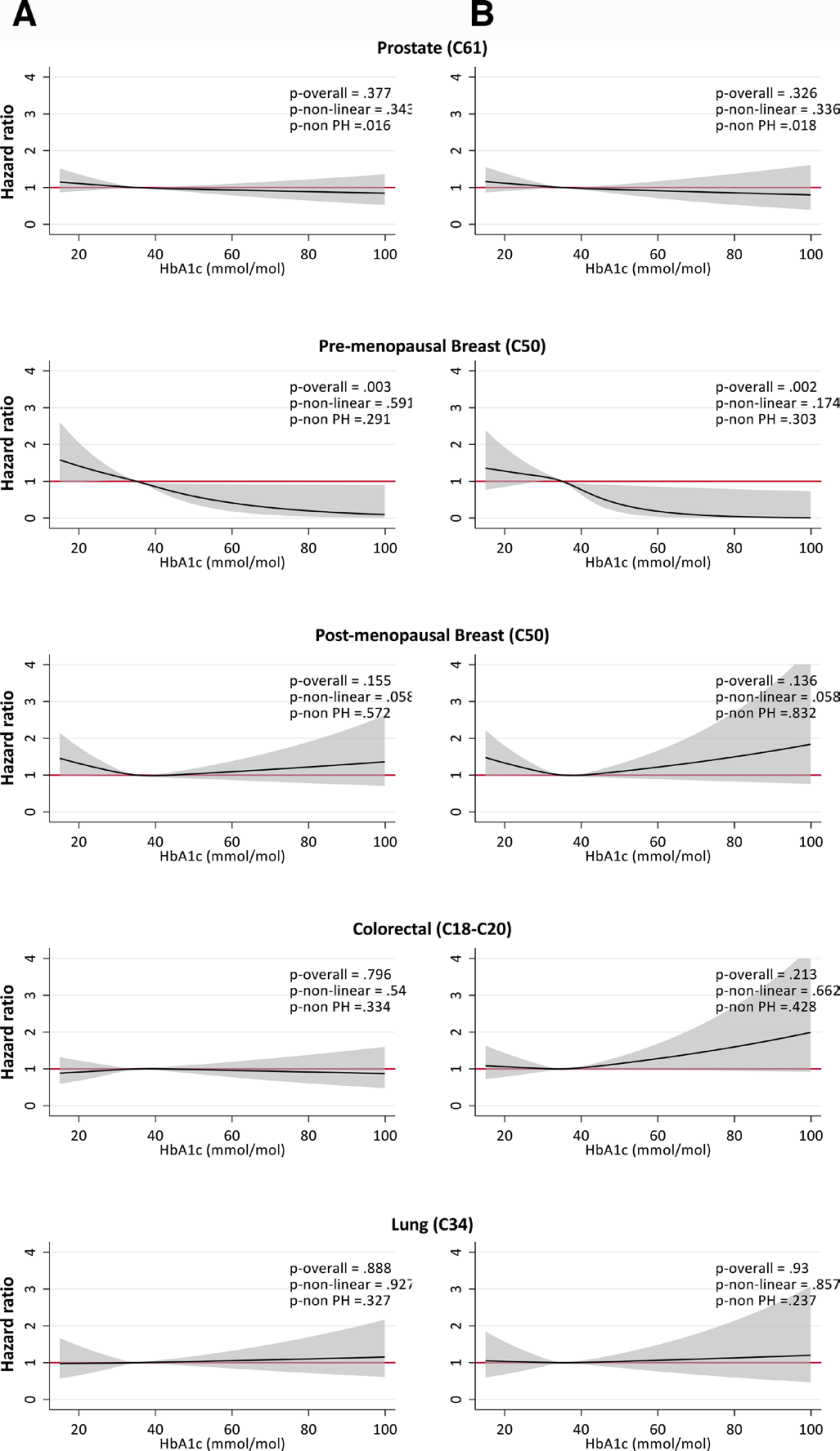
Associations between glycated hemoglobin (HbA1c) and incidence of common cancers in the UK Biobank. HRs from Cox proportional hazards models, adjusted for age at study entry, sex (except sex-specific cancers), ethnicity, deprivation, body mass index, physical activity, cardiovascular disease, diagnosed diabetes, smoking status, alcohol consumption, use of oral contraceptives and hormone replacement therapy (postmenopausal breast only), and processed meat and fruit and vegetable intake (colorectal only). Reference HbA1c=5.4% (35 mmol/mol). Results shown including (left) and excluding (right) those with diagnosed diabetes at baseline. Significance tests include p overall for test of overall association, p non-linear for test of linearity, p non-PH for test of proportional hazards.

Overall, there was no clear evidence of an association between HbA1c and incidence of prostate, colorectal, and lung cancer after adjustment for a comprehensive range of confounders (figure 2A). There was evidence of increased risk of premenopausal breast cancer at lower levels of HbA1c (HR 1.27, 95% CI 1.00 to 1.60 for 25 vs 35 mmol/mol) and decreased risk at higher levels of HbA1c (HR 0.71, 95% CI 0.54 to 0.94 for 45 vs 35 mmol/mol; HR 0.50, 95% CI 0.26 to 0.93 for 55 vs 35 mmol/mol). There was weak evidence of an association between lower HbA1c and increased risk of postmenopausal breast cancer (HR 1.19, 95% CI 0.99 to 1.43 for 25 vs 35 mmol/mol). Results were broadly similar when restricting to individuals without a diabetes diagnosis or exposure to glucose-lowering medication at baseline (figure 2B). In this subgroup analysis, there was some suggestion of an increased risk of colorectal cancer with increasing HbA1c (HR 1.21, 95% CI 0.98 to 1.50 for 55 vs 35 mmol/mol), though there were only 24 events in this range of HbA1c.

Among the 12 less common cancers investigated, with the exception of pancreatic and uterine cancer, there was no clear pattern of association between HbA1c and cancer incidence, although CIs were wide due to limited numbers of events (online supplementary figure S1). There was relatively strong evidence that HbA1c was positively associated with pancreatic cancer, whereby low HbA1c was associated with lower cancer risk (HR 0.39, 95% CI 0.25 to 0.62 for 25 vs 35 mmol/mol) and high HbA1c was associated with elevated cancer risk (HR 1.55, 95% CI 1.22 to 1.98 for 55 vs 35 mmol/mol). There was also evidence that low HbA1c was associated with lower risk of uterine cancer (HR 0.52, 95% CI 0.34 to 0.79 for 25 vs 35 mmol/mol). These findings remained when excluding those with a diabetes diagnosis at baseline (online supplementary figure S2).

10.1136/bmjdrc-2020-001600.supp2 Supplementary data

### Sensitivity analyses

For the majority of cancers, there was very little qualitative change after excluding individuals with smoking history at baseline, though in some cases, such as the inverse association with premenopausal cancer, statistical significance was lost (online supplementary figure S3). Fitting the model separately by time since study entry did not suggest that any of the observed results were influenced by reverse causation (online supplementary figures S4-S5). Adjusting for diagnosed diabetes at baseline consistently had a large impact on the observed associations with all primary cancer outcomes, in addition to age adjustment for prostate and colorectal cancer and smoking and alcohol adjustment for lung cancer (online supplementary figures S6-S10). All other confounder adjustments tended to make relatively minor, incremental differences. When comparing cancer risk in people with and without diagnosed type 2 diabetes at baseline, we found evidence of decreased risk of prostate cancer (HR 0.75, 95% CI 0.66 to 0.86) and increased risk of colorectal cancer (HR 1.23, 95% CI 1.05 to 1.44), pancreatic cancer (HR 1.59, 95% CI 1.21 to 2.09), uterine cancer (HR 1.52, 95% CI 1.13 to 2.04), and bladder cancer (HR 1.60, 95% CI 1.21 to 2.11) (online supplementary figure S11). In post hoc analyses, while variance in estimates increased due to the lower number of events, the shape of the association between HbA1c and premenopausal breast cancer observed in primary analyses remained after adjusting for age at first live birth (online supplementary figure S12). Finally, models using age as the timescale did not change conclusions from models using time since baseline as the timescale (online supplementary figure S13).

## Conclusions

In this study of 378 253 adults with 21 172 incident cancer events over an average 7 years of follow-up, we found no consistent evidence that higher HbA1c was associated with incident cancer risk among nearly all of the 16 cancer types investigated apart from pancreatic cancer. A novel finding was an inverse association between HbA1c and premenopausal breast cancer, which persisted when people with diagnosed diabetes or on glucose-lowering medications were excluded. This study is the largest study to date to investigate the association between HbA1c and multiple specific cancers adjusting for a wide range of demographic, lifestyle, and clinical factors. There was no suggestion that any observed associations (or lack thereof) with cancer incidence were explained by reverse causality.

We examined the associations between cancer risk and having a diagnosis of type 2 diabetes at baseline and found positive associations with colorectal, pancreatic, uterine and bladder cancer, and a negative association with prostate cancer. This is consistent with previous research, alleviating concerns around UK Biobank participants being healthier than the general population.^13^ Interpretation of previous studies of individual cancer incidence across the entire glycemic spectrum has been limited by small numbers. Much of our understanding of this association therefore comes from dichotomized comparisons of people with and without diagnosed type 2 diabetes. We were able to take these analyses further, exploring associations across the full glycemic range. Exclusion of people with diagnosed diabetes or prescribed metformin for other indications addresses the uncertainties around effects of glucose-lowering medication.^3^ For the most part, this had little impact on associations. Thus, apart from known associations with pancreatic cancer,^14^ we were not able to demonstrate any consistent link between higher HbA1c and risk for cancer that would support a hyperglycemia associated mechanism for cancer risk.

We found a novel inverse association between HbA1c and premenopausal breast cancer, whereby increased HbA1c was associated with decreased risk of cancer, that were not attributed to differences in demographic characteristics, BMI, or lifestyle factors. There have been previous suggestions that glucose-lowering therapies, particularly metformin, are associated with lower risk of breast cancer though concerns over bias were raised.^15 16^ Subsequent studies have found no such evidence.^17^ In our current analysis, we demonstrated the relationship between HbA1c and premenopausal breast cancer could not be attributed to glucose-lowering therapy since the association was also found in a model excluding those with diagnosed diabetes or on metformin. Previous studies have found that younger maternal age at first birth increases risk of hyperglycemia and diabetes.^18 19^ In contrast, older maternal age at first birth is one of the key risk factors for premenopausal breast cancer, a factor that does not appear to contribute to postmenopausal cancer.^20^ Low normal glycemia could be acting as a proxy for older age at first birth, potentially accounting for the inverse association between hyperglycemia and premenopausal breast cancer risk. However, after adjusting estimates for maternal age at first live birth in post hoc analyses, the inverse association observed in primary analyses remained. Another possible mechanism could be that women with diabetes or higher BMI (ie, those with elevated HbA1c) are more engaged with healthcare services resulting in earlier detection of breast cancer; however, this information is not available in UK Biobank.

We had several incidental findings in sensitivity analyses. For example, after excluding individuals with diagnosed diabetes or on glucose-lowering medications, there was a suggestion that increased HbA1c was associated with elevated risk of incident stomach cancer. This finding is consistent with a previous study of 2603 individuals that accounted for confounding factors including BMI and alcohol intake^21^ but contradicts null or potentially underpowered findings from two larger studies by Travier *et al*^22^ and Miao Jonasson *et al*^23^ (n=46 575 and n=25 276, respectively). Caution should be used when interpreting this and findings from other sensitivity analyses since they were not observed in primary analyses. There remains the possibility that the observations occurred simply by chance due to the number of comparisons we performed in sensitivity analyses or perhaps due to collider bias introduced by excluding populations.

The most recent systematic review of associations between HbA1c and cancer risk included 19 studies,^4^ the largest of which analyzed 46 575 individuals with 634 cancer events.^22^ While the authors found no evidence of an association between HbA1c and any of the cancers also included in the present study, the number of site-specific cancer events were low. A more recent study by Goto *et al*^24^ followed 29 629 individuals with HbA1c measurements of whom only 6% had an existing diabetes diagnosis. The authors found an overall increased risk of cancer with increasing HbA1c, which was not observed after excluding events in the first 3 years of follow-up. A large study by Dankner *et al*^7^ included 440 000 individuals with diagnosed diabetes from a large insurance database. With over 26 000 cancer events, they found no association between HbA1c and overall cancer risk but did observe a positive association with pancreatic cancer and negative association with prostate cancer. We confirm an association with pancreatic cancer; however, we show that any protective effect of HbA1c on prostate cancer is lost after adjustment, namely for diagnosed diabetes. Danker *et al* were not able to adjust for variables included in the UK Biobank, including BMI, physical activity, and underlying cardiovascular disease, and only included individuals with diagnosed diabetes thus effects may be confounded by exposure to glucose-lowering therapies or some other unmeasured factor.

Our study has several key strengths, particularly a large sample size, the inclusion of individuals with and without diagnosed diabetes and as such not exposed to any glucose-lowering medications, a long follow-up window, and highly detailed covariate data with small proportions of missingness. We ascertained cancer outcomes from highly reliable linked UK cancer registry data covering all treated cancers in the National Health Service. We also acknowledge some important limitations. We approximated menopausal status, meaning that there may be some misclassification of premenopausal or postmenopausal breast cancers; however, previous studies have suggested that such proxies produce valid incidence rates.^10^ The overall healthier population contributing to the UK Biobank^13^ meant that we observed relatively low cancer incidence rates, and as such, the power of our analysis was likely affected. Although the underlying incidence rates and exposure distributions may not be representative of the UK population, the associations observed between HbA1c and cancer incidence are unlikely to be biased.^25^ This is supported by our sensitivity analysis replicating associations between type 2 diabetes and cancer incidence that are consistent with a recent umbrella review.^2^ Lastly, it is possible that although we adjusted for a wide range of confounders, some residual confounding may remain. We were also unable to look at the effect of medication use in those with diagnosed diabetes as we lacked longitudinal prescription data.

In conclusion, apart from pancreatic cancer, we did not demonstrate any independent positive association between HbA1c and risk for cancer in this large sample of UK adults. We identified an inverse association between HbA1c and premenopausal breast cancer unlikely to be attributed to antidiabetic therapies. These findings suggest that concerns around the potential for a cancer-inducing, direct effect of hyperglycemia may be misplaced. Future research should explore other potential mechanisms that have been hypothesized, including genetic risk factors and chronic inflammation.
