# Supplementary material for: Risk of 16 cancers across the full glycemic spectrum: a population-based cohort study using the UK Biobank
Source: BMJ Open Diabetes Res Care. 2020 Aug 27;8(1):e001600. doi: 10.1136/bmjdrc-2020-001600 (PMC7454242; doi:10.1136/bmjdrc-2020-001600)
Supplement: Supplementary data [file bmjdrc-2020-001600supp001.pdf]

Table S1 Baseline characteristics of 360,768 participants without diagnosed diabetes or exposure to glucose lowering medications

	**<6% or <42 mmol/mol**	**6-6.4% or 42-47 mmol/mol**	**≥6.5% or ≥48 mmol/mol**
**N**	346,398	11,829	2,541
**Age at baseline assessment, years (Median (IQR))**	57.0 (49.0, 63.0)	61.0 (56.0, 65.0)	60.0 (53.0, 64.0)
**Female (N %)**	188,776 (54.5)	6,030 (51.0)	965 (38.0)
**Ethnicity**			
White European	333,012 (96.1)	10,392 (87.9)	2,178 (85.7)
South Asian	3,750 (1.1)	409 (3.5)	132 (5.2)
African Caribbean	3,467 (1.0)	541 (4.6)	124 (4.9)
Mixed or other	6,169 (1.8)	487 (4.1)	107 (4.2)
**Townsend deprivation index quintile**			
Least deprived	73,899 (21.3)	2,061 (17.4)	415 (16.3)
2nd least deprived	72,563 (20.9)	2,170 (18.3)	398 (15.7)
Median deprivation level	71,034 (20.5)	2,204 (18.6)	455 (17.9)
2nd most deprived	68,352 (19.7)	2,526 (21.4)	535 (21.1)
Most deprived	60,550 (17.5)	2,868 (24.2)	738 (29.0)
**Smoking status**			
Never Smoker	243,388 (70.3)	6,953 (58.8)	1,489 (58.6)
Former Smoker	79,396 (22.9)	3,377 (28.5)	768 (30.2)
1-9 CPD	5,112 (1.5)	210 (1.8)	26 (1.0)
10-19 CPD	10,021 (2.9)	643 (5.4)	125 (4.9)
20-29 CPD	6,741 (1.9)	499 (4.2)	101 (4.0)
30+ CPD	1,740 (0.5)	147 (1.2)	32 (1.3)
**Alcohol use**			
Daily or almost daily	73,456 (21.2)	1,945 (16.4)	402 (15.8)
Three or four times a week	85,449 (24.7)	2,014 (17.0)	413 (16.3)
Once or twice a week	90,906 (26.2)	2,914 (24.6)	615 (24.2)
One to three times a month	38,257 (11.0)	1,539 (13.0)	340 (13.4)
Special occasions only	35,231 (10.2)	2,004 (16.9)	406 (16.0)
Never	23,099 (6.7)	1,413 (11.9)	365 (14.4)
**Days per week spent doing moderate physical activity**			
None	42,914 (12.4)	1,871 (15.8)	516 (20.3)
1-2	80,255 (23.2)	2,516 (21.3)	567 (22.3)
3-4	52,865 (15.3)	1,730 (14.6)	327 (12.9)
5+	170,364 (49.2)	5,712 (48.3)	1,131 (44.5)
**Days per week spent doing vigorous physical activity**			
None	121,134 (35.0)	5,277 (44.6)	1,271 (50.0)
1-2	107,528 (31.0)	3,213 (27.2)	640 (25.2)
3-4	50,105 (14.5)	1,319 (11.2)	250 (9.8)
5+	67,631 (19.5)	2,020 (17.1)	380 (15.0)
**Days per week walked for > 10 minutes**			
None	7,175 (2.1)	369 (3.1)	121 (4.8)

1-2	30,784 (8.9)	1,115 (9.4)	264 (10.4)
3-4	27,444 (7.9)	964 (8.1)	213 (8.4)
5+	280,995 (81.1)	9,381 (79.3)	1,943 (76.5)
**Days per week with intake of processed meats**			
None	32,631 (9.4)	910 (7.7)	171 (6.7)
<1	107,202 (31.0)	3,274 (27.7)	602 (23.7)
1	100,954 (29.1)	3,503 (29.6)	723 (28.5)
2-4	92,360 (26.7)	3,585 (30.3)	881 (34.7)
5+	13,251 (3.8)	557 (4.7)	164 (6.5)
**Number of fruits or vegetables consumed per day**			
None	10,622 (3.1)	558 (4.7)	145 (5.7)
1-2	60,948 (17.6)	2,238 (18.9)	571 (22.5)
3-4	101,538 (29.3)	3,465 (29.3)	737 (29.0)
5+	173,290 (50.0)	5,568 (47.1)	1,088 (42.8)
**BMI at baseline, kg/m^2 (Median (IQR))**	26.4 (23.9, 29.3)	29.5 (26.5, 33.2)	31.1 (28.0, 35.1)
**HbA1c at baseline, mmol/mol (Median (IQR))**	34.7 (32.4, 37.0)	43.4 (42.5, 44.7)	52.7 (49.6, 61.3)
**Comorbidities reported at baseline**			
Any cardiovascular disease	39,955 (11.5)	2,549 (21.5)	505 (19.9)
**Medications reported at baseline**			
Hormone replacement therapy	14,403 (4.2)	243 (2.1)	39 (1.5)
Oral contraceptives	5,612 (1.6)	50 (0.4)	21 (0.8)

Abbreviations: HbA1c, glycated haemoglobin; IQR, interquartile range; CPD, cigarettes smoked per day; BMI, body mass index in kilograms per square metre (kg/m^2)

Table S2 Number of events and age-standardised incidence rates among participants without diagnosed diabetes or exposure to metformin at baseline, by HbA1c category

	<6% or <42 mmol/mol		6-6.4% or 42-47 mmol/mol		≥6.5% or ≥48 mmol/mol	
	N	Rate/1000 PY (95% CI)	N	Rate/1000 PY (95% CI)	N	Rate/1000 PY (95% CI)
**Any malignant cancer***	18,740	8.63 (8.52 - 8.75)	911	10.27 (9.59 - 10.98)	175	9.84 (8.31 - 11.29)
**Primary outcomes**						
Prostate (C61) †	4,055	4.11 (3.98 - 4.24)	177	3.90 (3.33 - 4.48)	38	3.44 (2.48 - 4.61)
Pre-menopausal Breast (C50) ‡	1,001	1.11 (1.04 - 1.18)	3	0.38 (0.07 - 0.75)	2	1.09 (0.56 - 1.71)
Post-menopausal Breast (C50) ‡	2,570	3.34 (3.06 - 3.65)	107	6.82 (2.05 - 17.28)	22	3.04 (1.95 - 4.85)
Colorectal (C18-C20)	2,170	1.00 (0.96 - 1.05)	99	1.08 (0.88 - 1.32)	24	1.42 (0.85 - 2.03)
Lung (C34)	1,359	0.63 (0.61 - 0.67)	125	1.38 (1.14 - 1.63)	15	0.83 (0.44 - 1.27)
**Secondary outcomes**						
Oesophageal (C15)	387	0.18 (0.16 - 0.20)	26	0.29 (0.18 - 0.41)	6	0.33 (0.11 - 0.62)
Stomach (C16)	265	0.12 (0.11 - 0.14)	23	0.23 (0.14 - 0.33)	6	0.40 (0.11 - 0.79)
Pancreatic (C25)	488	0.23 (0.21 - 0.25)	36	0.47 (0.32 - 0.66)	12	0.65 (0.31 - 1.03)
Melanoma (C43)	1,162	0.53 (0.50 - 0.56)	47	0.49 (0.34 - 0.64)	8	0.45 (0.18 - 0.77)
Uterus (C54-55) ‡	550	0.46 (0.43 - 0.51)	38	0.94 (0.63 - 1.27)	4	0.55 (0.13 - 1.17)
Ovarian (C56) ‡	435	0.37 (0.34 - 0.41)	12	0.40 (0.14 - 0.74)	4	0.68 (0.00 - 1.45)
Kidney (C64)	491	0.23 (0.21 - 0.25)	30	0.37 (0.23 - 0.51)	6	0.37 (0.11 - 0.68)
Bladder (C67)	386	0.18 (0.16 - 0.20)	17	0.17 (0.09 - 0.26)	5	0.27 (0.06 - 0.55)
Non-Hodgkin Lymphoma (C82-85)	819	0.38 (0.35 - 0.41)	42	0.44 (0.31 - 0.60)	4	0.21 (0.05 - 0.44)
Multiple Myeloma (C90)	317	0.15 (0.13 - 0.16)	15	0.16 (0.07 - 0.25)	4	0.23 (0.05 - 0.48)
Leukaemia (C91-94)	477	0.22 (0.20 - 0.24)	30	0.33 (0.22 - 0.45)	2	0.11 (0.00 - 0.28)

NB. Standardised to the UK Biobank population, percentile confidence intervals obtained via 500 bootstrap replications.
Abbreviations: HbA1c, glycated haemoglobin; PY, person-years; CI, confidence interval
*Excluding non-melanoma skin cancer (C44)
†Males only
‡Females only

Table S3 Number of events and age-standardised incidence rates including first six months after baseline, by HbA1c category

	<6% or <42 mmol/mol		6-6.4% or 42-47 mmol/mol		≥6.5% or ≥48 mmol/mol	
	N	Rate/1000 PY (95% CI)	N	Rate/1000 PY (95% CI)	N	Rate/1000 PY (95% CI)
**Any malignant cancer***	20,118	8.51 (8.40 - 8.63)	1,322	10.40 (9.84 - 10.99)	969	9.76 (9.12 - 10.38)
**Primary outcomes**						
Prostate (C61) †	4,350	4.02 (3.90 - 4.13)	247	3.49 (3.00 - 3.97)	193	2.80 (2.42 - 3.22)
Pre-menopausal Breast (C50) ‡	1,400	3.29 (3.01 - 3.56)	14	6.41 (1.95 - 15.72)	19	3.04 (1.98 - 4.86)
Post-menopausal Breast (C50) ‡	2,786	1.06 (1.00 - 1.12)	143	0.55 (0.26 - 0.85)	92	0.91 (0.52 - 1.31)
Colorectal (C18-C20)	2,329	0.99 (0.95 - 1.03)	162	1.25 (1.04 - 1.46)	127	1.32 (1.10 - 1.54)
Lung (C34)	1,428	0.61 (0.58 - 0.64)	169	1.31 (1.08 - 1.52)	87	0.85 (0.67 - 1.05)
**Secondary outcomes**						
Oesophageal (C15)	412	0.18 (0.16 - 0.19)	40	0.30 (0.21 - 0.40)	41	0.40 (0.28 - 0.53)
Stomach (C16)	291	0.12 (0.11 - 0.14)	34	0.24 (0.16 - 0.32)	21	0.24 (0.14 - 0.36)
Pancreatic (C25)	507	0.22 (0.20 - 0.23)	57	0.48 (0.33 - 0.62)	57	0.57 (0.41 - 0.74)
Melanoma (C43)	1,254	0.52 (0.05 - 0.55)	66	0.50 (0.37 - 0.64)	41	0.41 (0.29 - 0.54)
Uterus (C54-55) ‡	601	0.47 (0.44 - 0.51)	52	0.99 (0.72 - 1.31)	33	0.93 (0.61 - 1.27)
Ovarian (C56) ‡	465	0.37 (0.33 - 0.40)	17	0.37 (0.17 - 0.65)	17	0.49 (0.29 - 0.76)
Kidney (C64)	526	0.22 (0.20 - 0.24)	46	0.39 (0.27 - 0.52)	42	0.42 (0.31 - 0.57)
Bladder (C67)	424	0.18 (0.16 - 0.20)	33	0.21 (0.14 - 0.29)	46	0.45 (0.32 - 0.59)
Non-Hodgkin Lymphoma (C82-85)	876	0.37 (0.35 - 0.40)	63	0.46 (0.34 - 0.59)	39	0.42 (0.30 - 0.56)
Multiple Myeloma (C90)	325	0.14 (0.12 - 0.15)	19	0.14 (0.08 - 0.21)	18	0.18 (0.10 - 0.28)
Leukaemia (C91-94)	513	0.22 (0.20 - 0.24)	44	0.34 (0.23 - 0.45)	23	0.23 (0.14 - 0.33)

NB. Standardised to the UK Biobank population, percentile confidence intervals obtained via 500 bootstrap replications.

Abbreviations: HbA1c, glycated haemoglobin; PY, person-years; CI, confidence interval

*Excluding non-melanoma skin cancer (C44)

†Males only

‡Females only
