# Supplementary material for: Risk of 16 cancers across the full glycemic spectrum: a population-based cohort study using the UK Biobank
Source: BMJ Open Diabetes Res Care. 2020 Aug 27;8(1):e001600. doi: 10.1136/bmjdrc-2020-001600 (PMC7454242; doi:10.1136/bmjdrc-2020-001600)

Figure S1 Associations between glycated haemoglobin (HbA1c) and incidence of secondary cancer outcomes

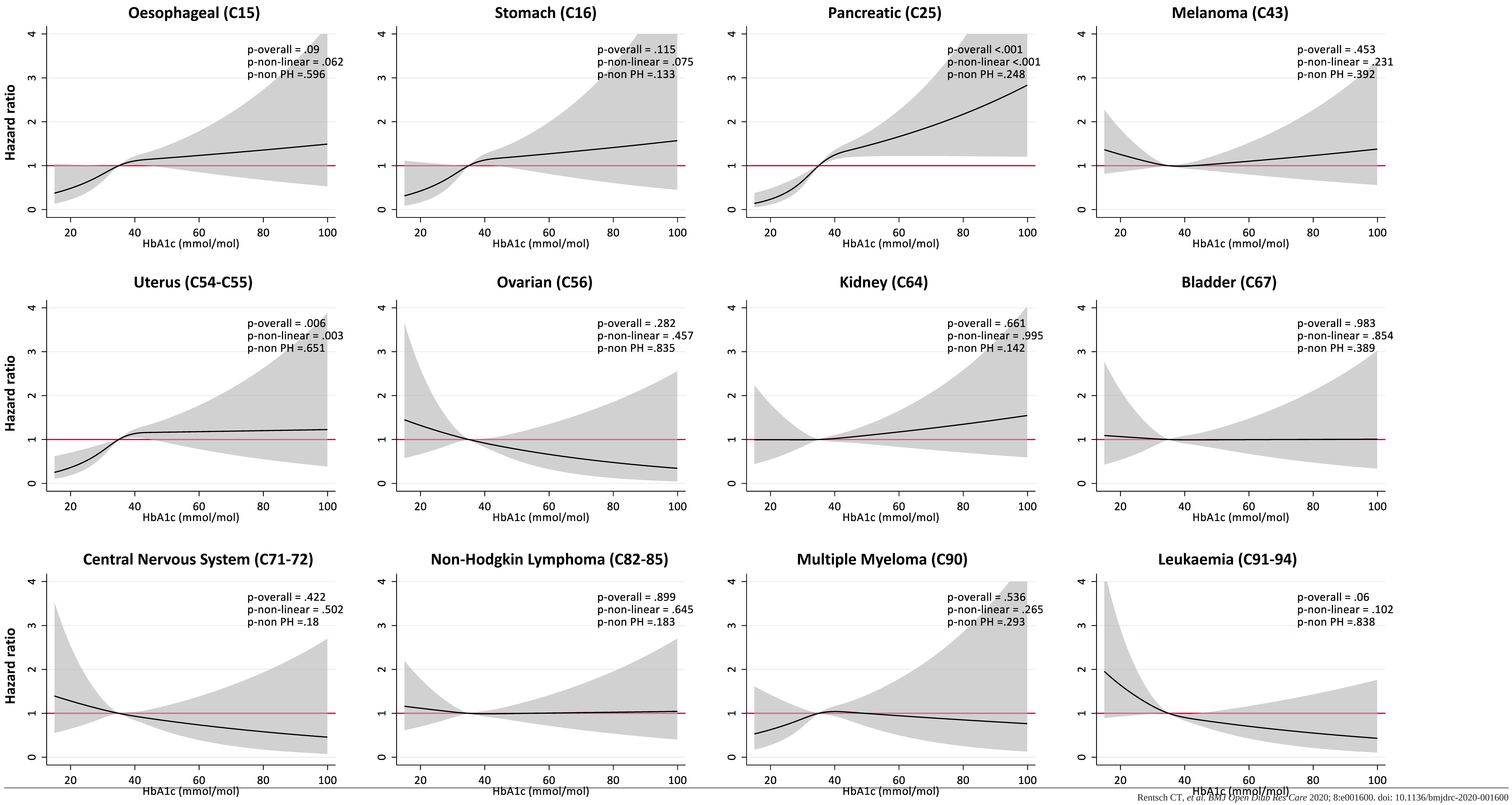

Figure S2 Associations between glycated haemoglobin (HbA1c) and incidence of secondary cancer outcomes, excluding participants who reported diabetes diagnosis or metformin exposure at baseline

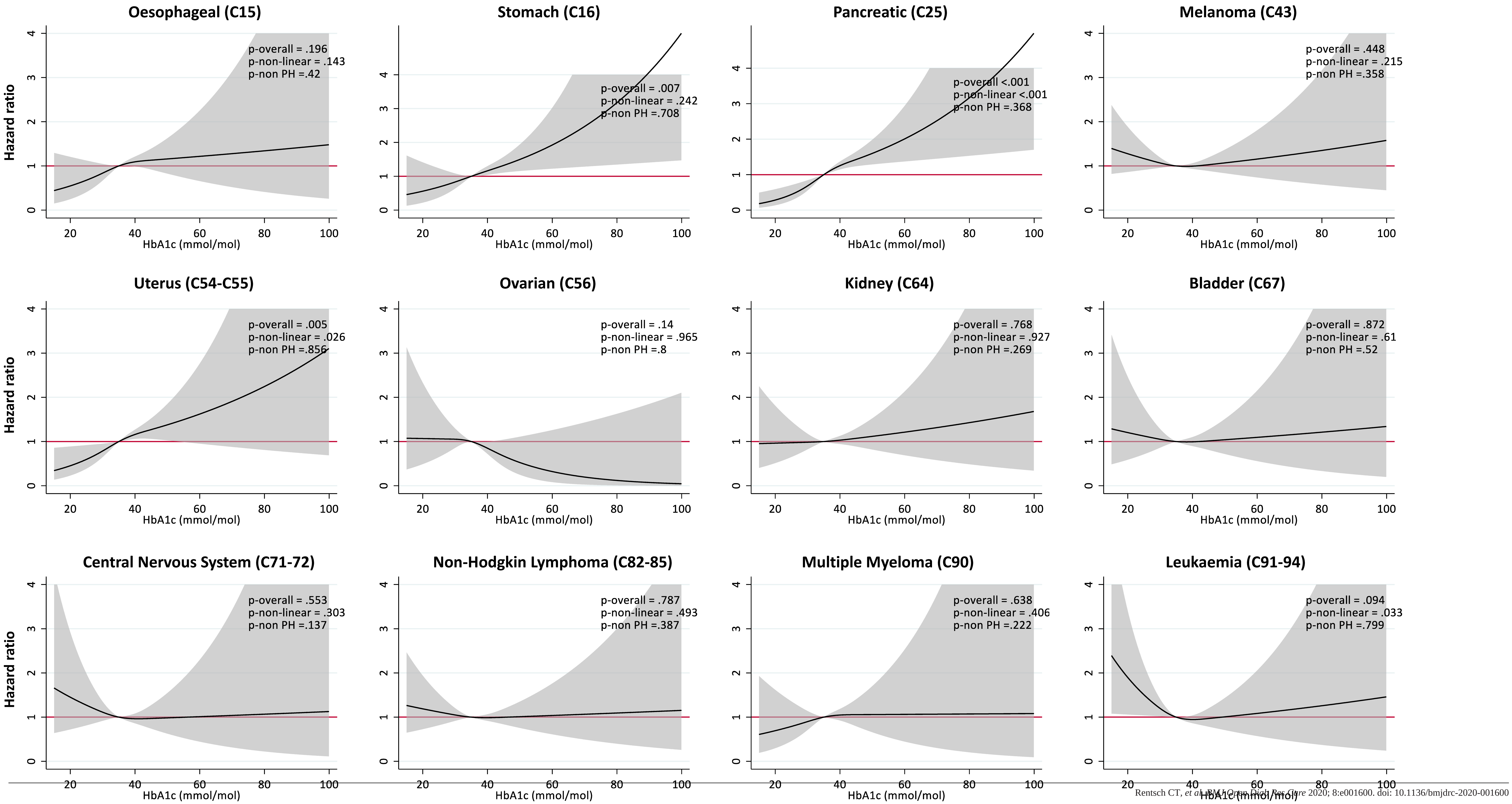

Figure S3 Associations between glycated haemoglobin (HbA1c) and incidence of 16 cancers, excluding participants with smoking history

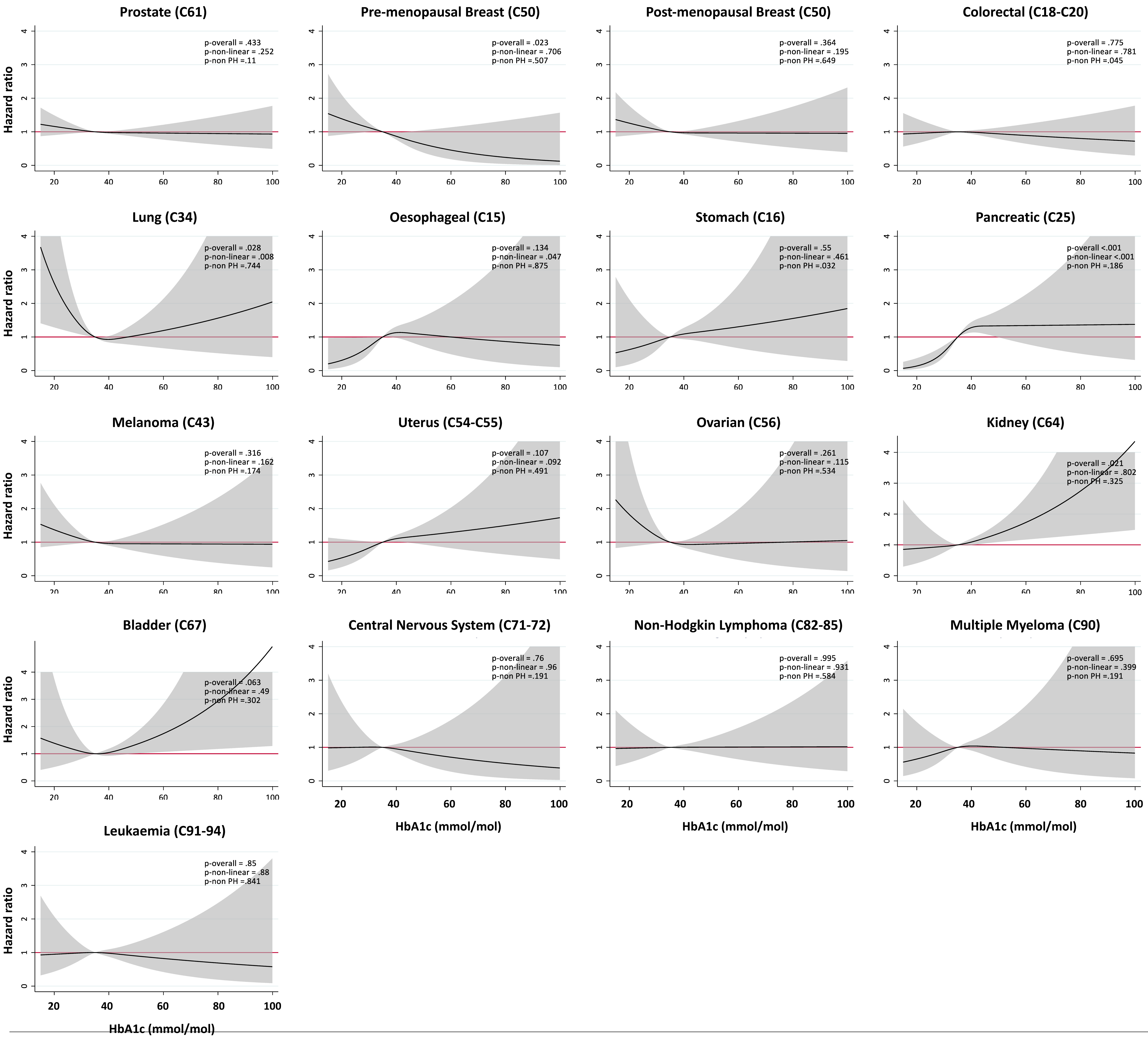

Figure S4 Associations between glycated haemoglobin (HbA1c) and incidence of primary cancer outcomes, by time since baseline

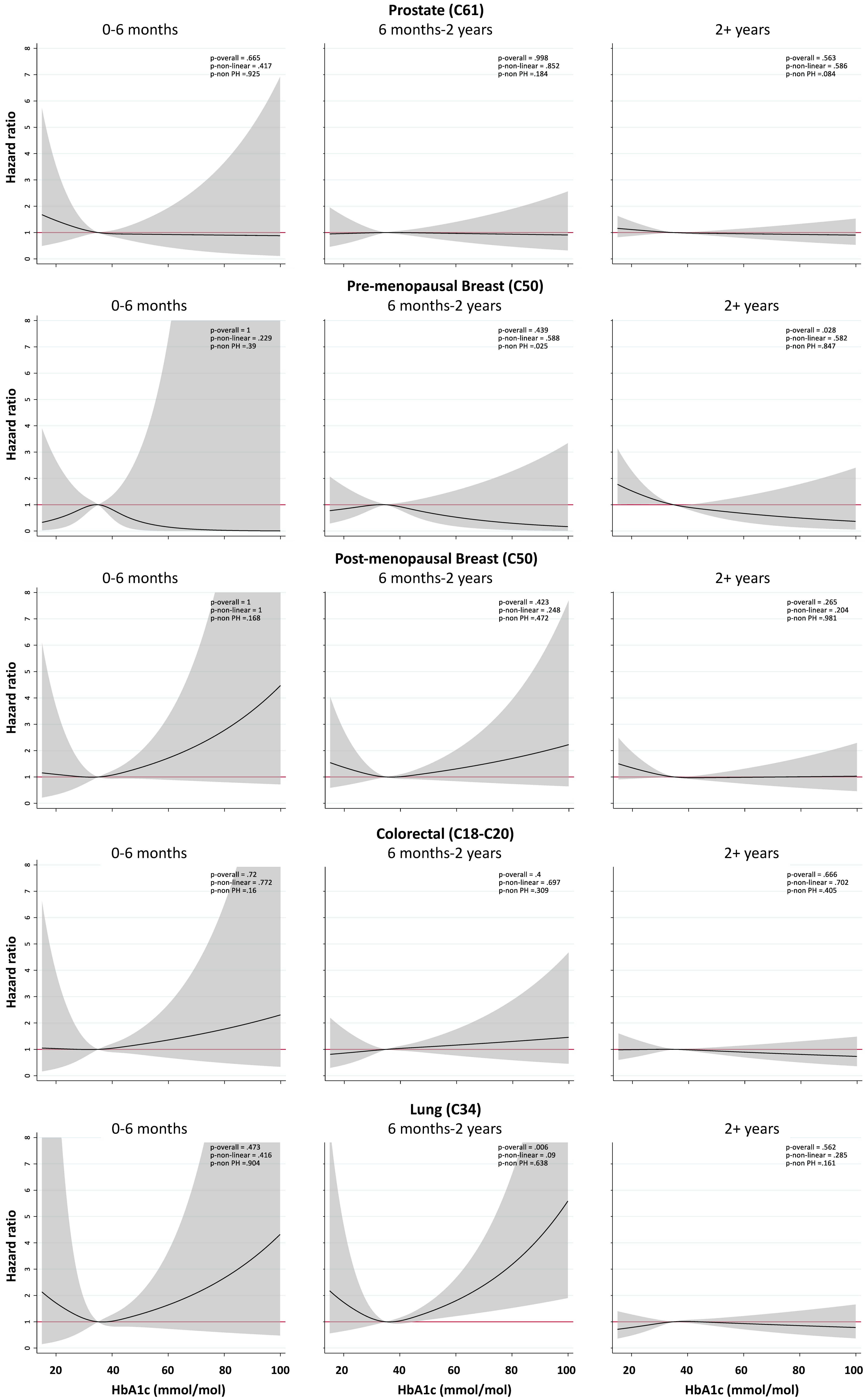

Figure S5 Associations between glycated haemoglobin (HbA1c) and incidence of primary cancer outcomes, by time since baseline, excluding participants who reported diabetes diagnosis or metformin exposure at baseline

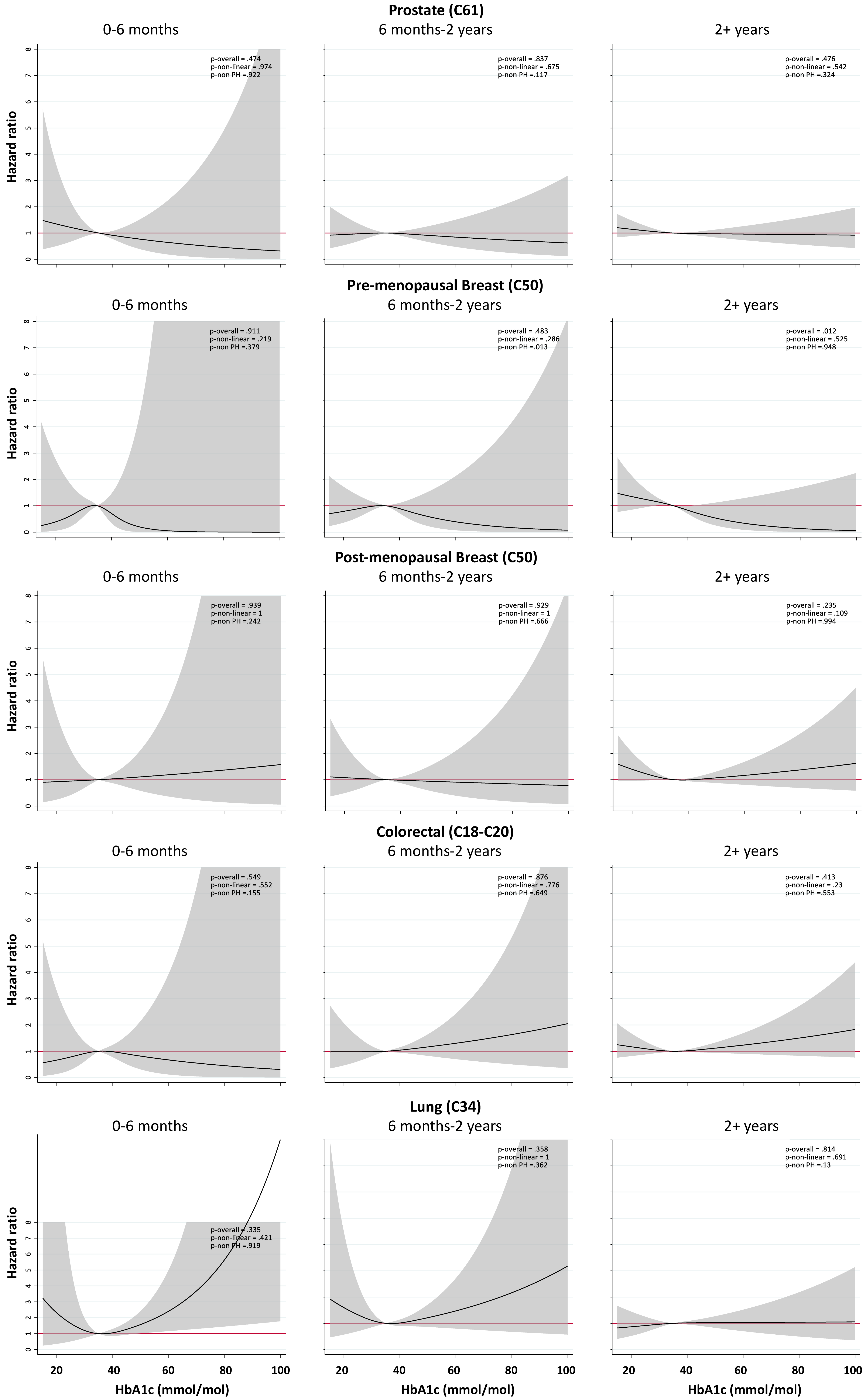

Figure S6 Prostate cancer and HbA1c at each additional confounder adjustment stage

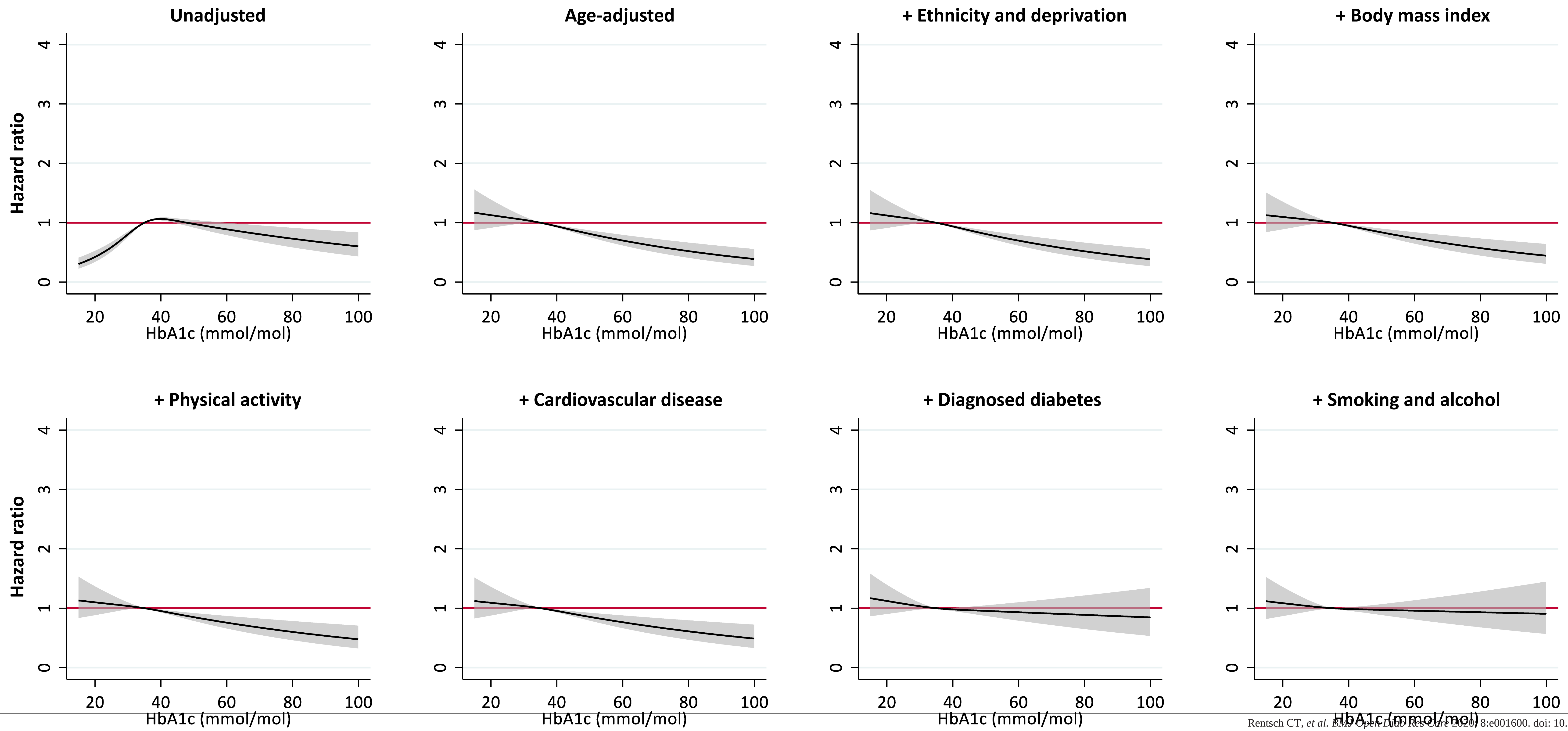

BMJ Publishing Group Limited (BMJ) disclaims all liability and responsibility arising from any reliance placed on this supplemental material which has been supplied by the author(s)

Figure S7 Pre-menopausal breast cancer and HbA1c at each additional confounder adjustment stage

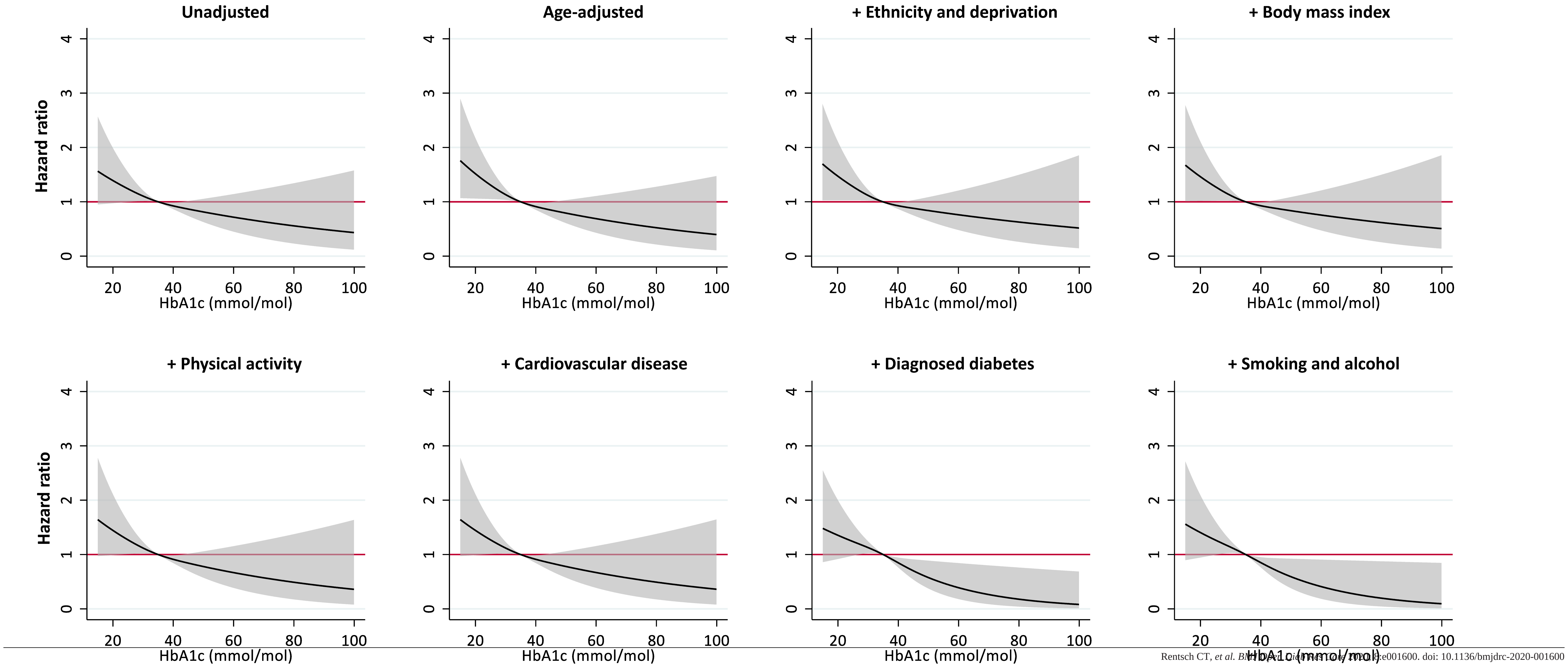

BMJ Publishing Group Limited (BMJ) disclaims all liability and responsibility arising from any reliance placed on this supplemental material which has been supplied by the author(s)

Figure S8 Post-menopausal breast cancer and HbA1c at each additional confounder adjustment stage

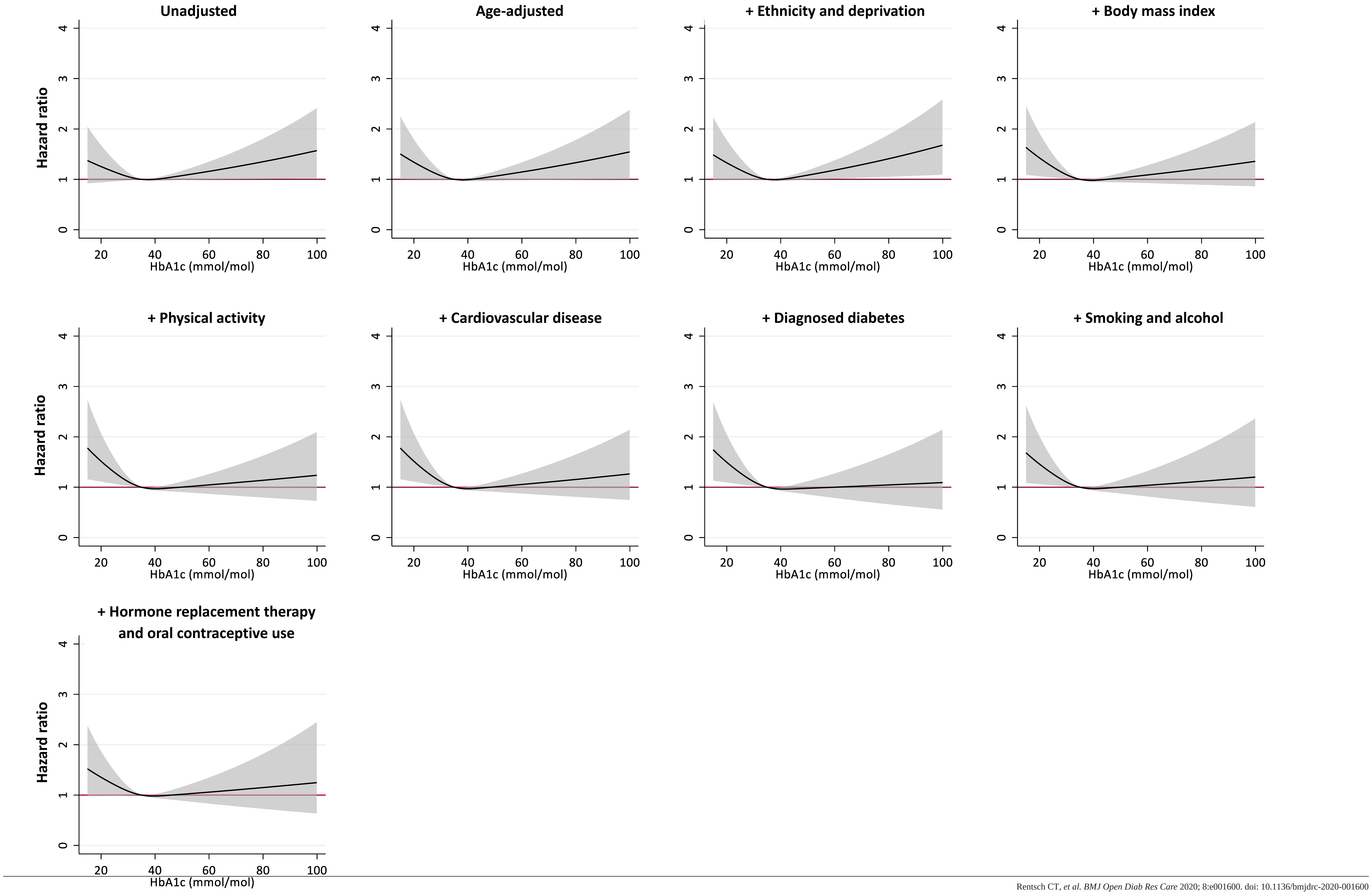

Figure S9 Colorectal cancer and HbA1c at each additional confounder adjustment stage

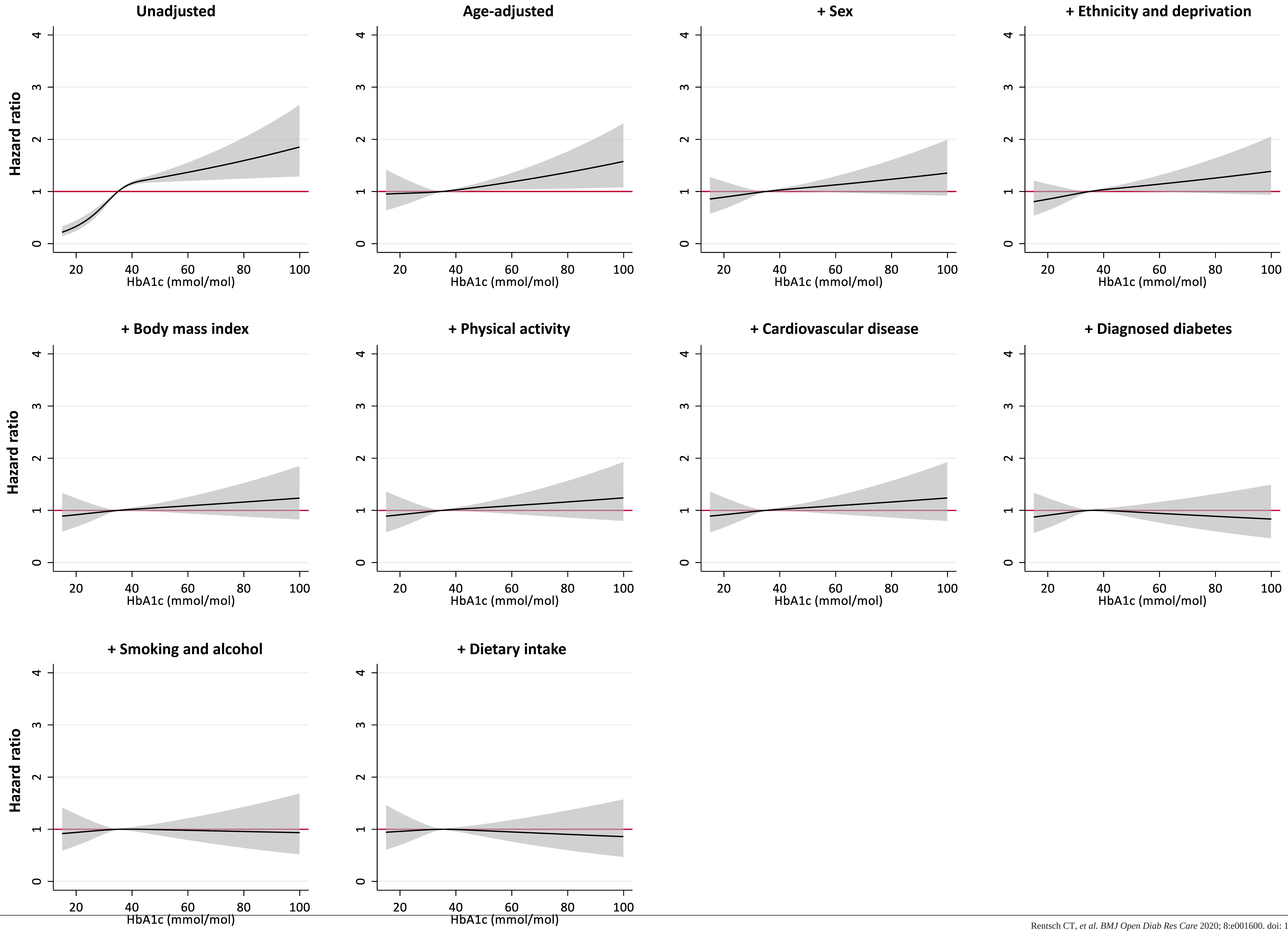

Figure S10 Lung cancer and HbA1c at each additional confounder adjustment stage

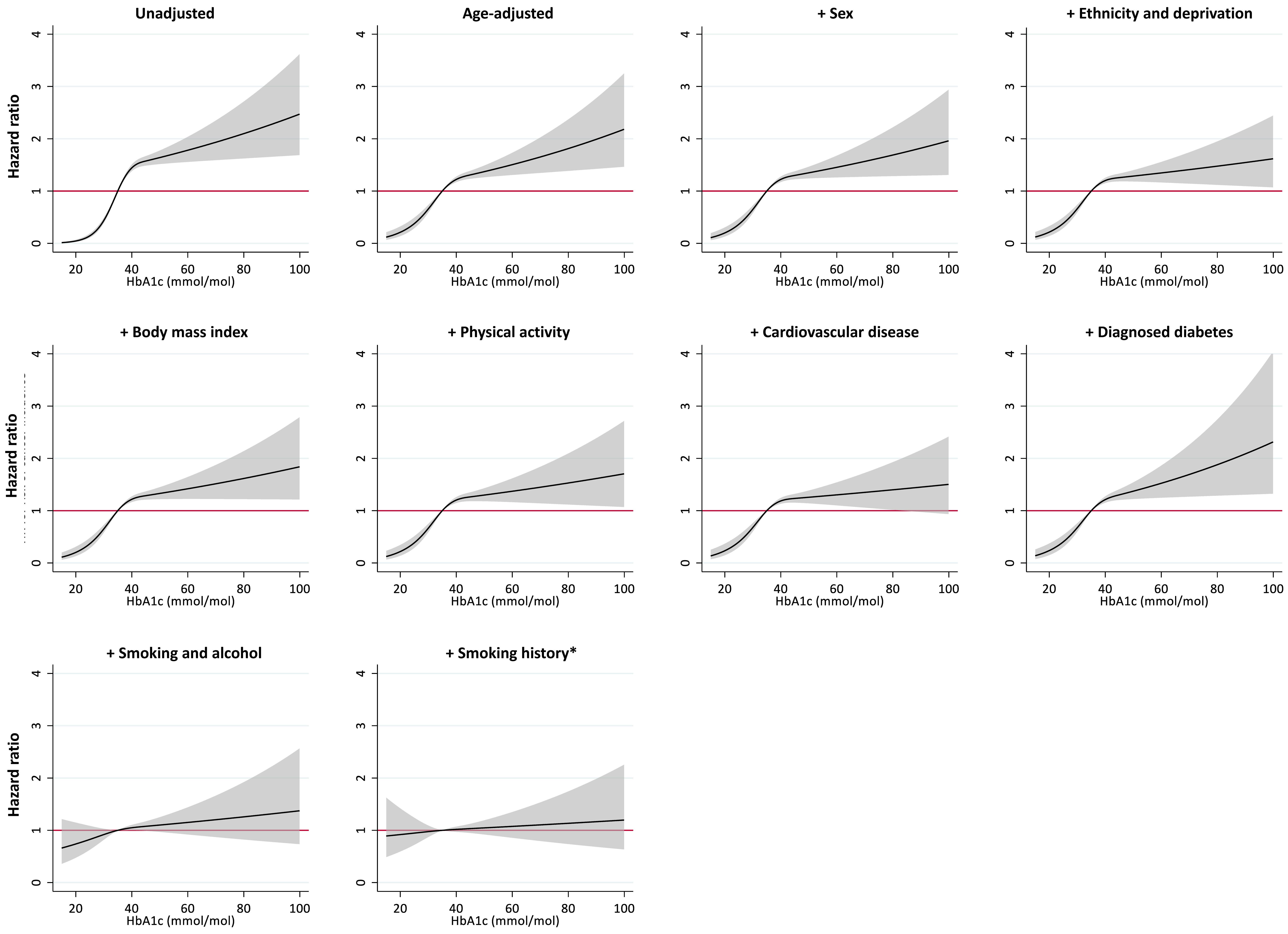

*Additionally adjusted for age at start and stop of smoking among former smokers and rate of smoking among current smokers

Figure S11 Association between baseline diagnosis of type 2 diabetes and cancer incidence in the UK Biobank

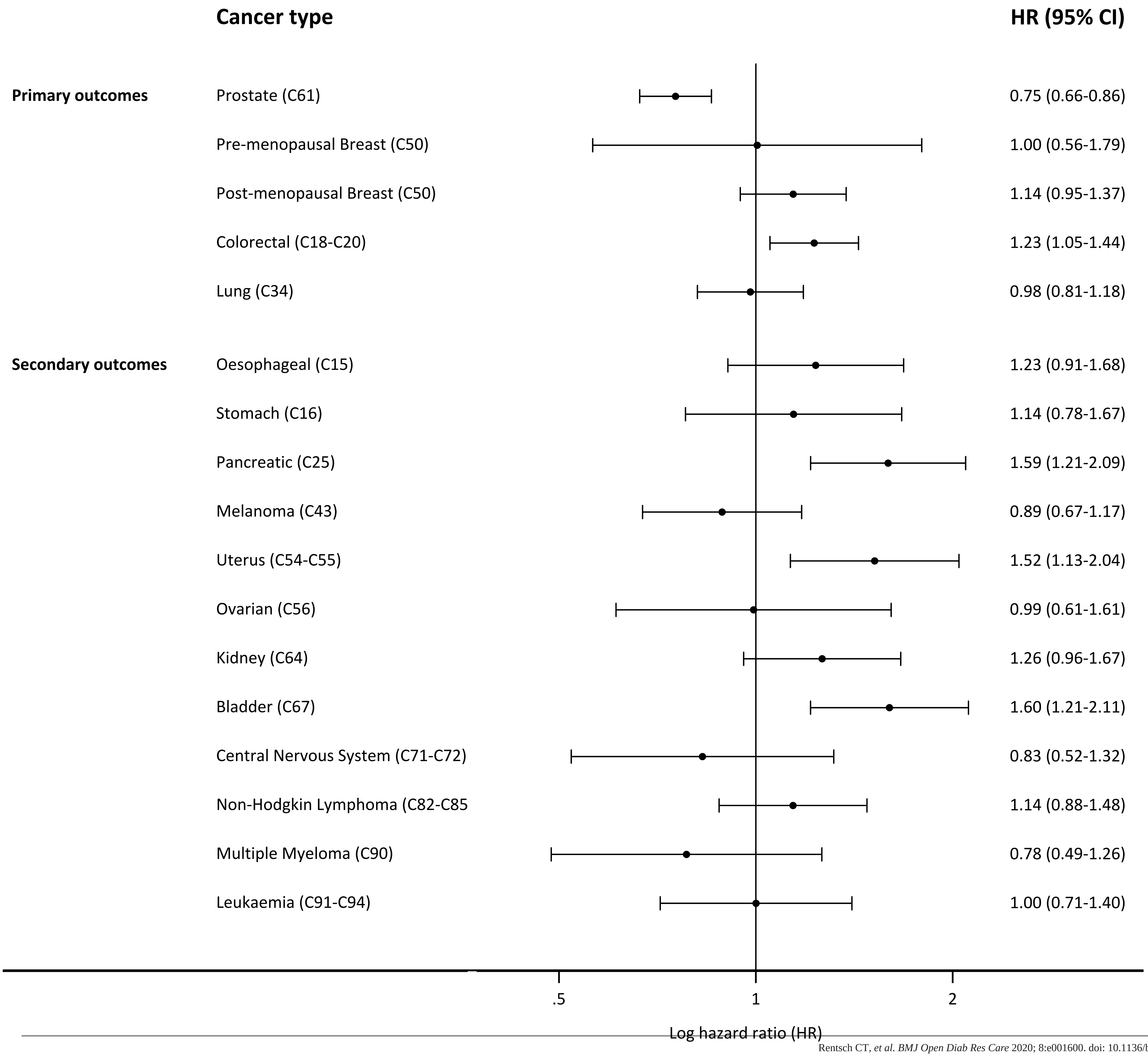

Figure S12 *Post hoc* analysis adjusting associations between HbA1c and pre-menopausal cancer (C50) for maternal age at first live birth

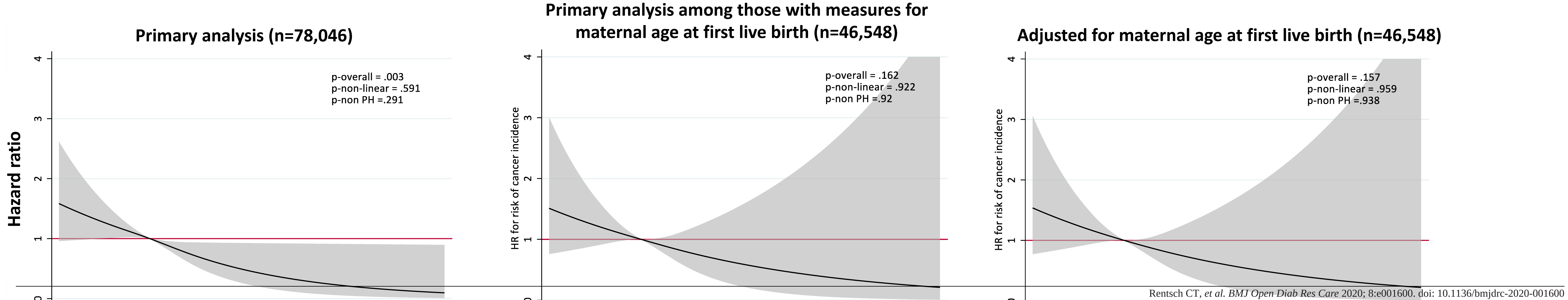

igure S13 Associations between glycated haemoglobin (HbA1c) and incidence of common ancers in the UK Biobank, using age as time scale

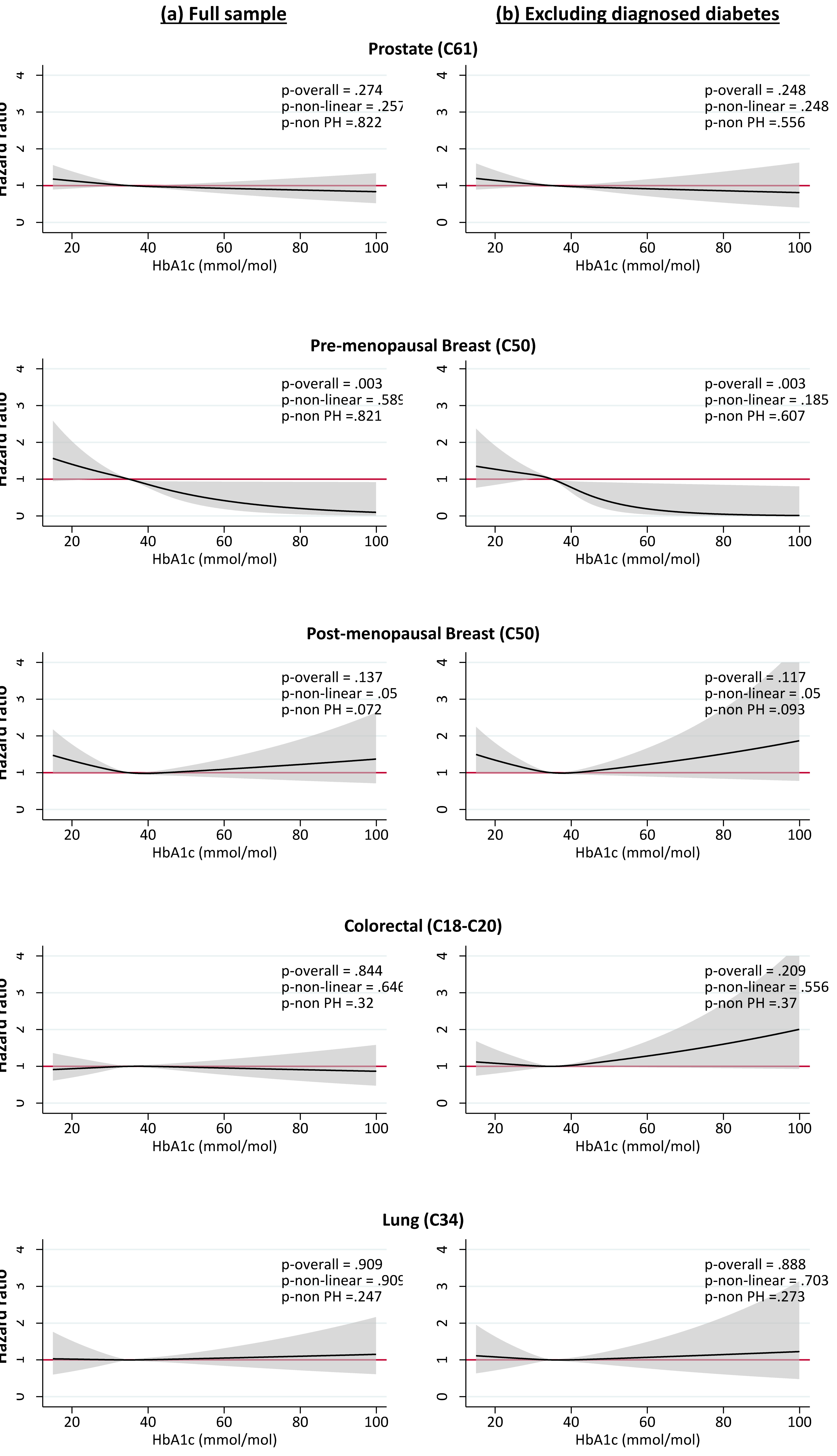

Supplement: Supplementary data [file bmjdrc-2020-001600supp002.pdf]
